# Pathogenic traits of *Salmonella* Montevideo in experimental infections *in vivo* and *in vitro*

**DOI:** 10.1038/srep46232

**Published:** 2017-04-07

**Authors:** Jonathan Lalsiamthara, John Hwa Lee

**Affiliations:** 1 College of Veterinary Medicine and Department of Bioactive Material Sciences, Chonbuk National University, Iksan Campus, Iksan 54596, Republic of Korea

## Abstract

*Salmonella* serovar Montevideo (SM) is frequently associated with human *Salmonella* infections and causes gastrointestinal disease, cases are common particularly among individuals who come in close contact with live poultry or poultry meat products. To characterize SM disease in chickens, the pathogenic traits and tissue predilections of the disease were investigated. Dissemination of fluorescent-tagged SM (JOL1575GFP) was monitored after oral and intramuscular mock infections of specific-pathogen-free chickens. The spleen was predominantly affected by intramuscular infection while the cecum, spleen, and minimally liver were affected by oral infection. No conspicuous illness was observed in infected birds, and histopathological examination showed minimal damage of the intestinal epithelium and splenic parenchyma though SM was readily isolated from these tissues. Levels of SM internalization by primary chicken peritoneal macrophages were similar to that of *Salmonella* Typhimurium. SM was more sensitive to chicken than rabbit serum complement killing. Internal egg contamination of SM mock infected layers also occurred at trace levels and lasted for a week after inoculation. This study also confirmed that SM infection in chickens is sub-clinical and asymptomatic, which suggests that latent asymptomatic carriers may excrete a large number of bacteria and transmit the pathogen by contaminating water or food sources.

Salmonellosis remains an important food-borne illness^1^. Recent years have seen a rise in human infections and outbreaks of a particular serotype, *Salmonella* Montevideo (SM), around the globe. It has been reported in the USA, Europe, Australia, and Asia including Korea and Japan^2^,^3^,^4^. In 2002, the first SM outbreaks were identified in Australia and New Zealand, and, since then, 68 infected individuals have been reported^5^. In 2010, an SM outbreak associated with a dietary food supplement was reported in Germany, with an unusual 15 SM infections being identified^6^. In the US, from 2009 to 2015, several SM outbreaks were reported, and the Centers for Disease Control and Prevention (CDC) recorded more than 500 cases during this period^2^,^7^,^8^. In several cases, the poultry sector was established as the source of SM. For instance, a multistate outbreak of human SM infections in 2012 in the US was linked to live poultry in backyard flocks^9^. In humans, non-typhoidal salmonellosis (NTS) is usually observed as localized entero-colitis, and the infected patients exhibit clinical signs of diarrhea, nausea, abdominal pain, mild fever, and chills^2^. Bacteremia due to multi-drug resistant SM was documented in a 3-year-old child^3^, and SM involvement has been reported in serous arthritis^10^, septic arthritis^11^, and acute myocarditis^12^. Cattle and sheep are commonly affected by SM infections, causing severe conditions, such as abortions, in sheep^13^. SM is readily isolated from cecum and cloacal swabs of infected chickens^14^. In most human SM infections, the likely source of the outbreak was live poultry or poultry products. While SM infection in humans is a gastro-intestinal disease, SM infection in chickens is not well documented. To date, it is unclear whether ‘salmonellosis’ is an appropriate term for chicken SM infection. Furthermore, contaminated eggs may transmit *Salmonella* to humans.

Our understanding of the pathogenic traits of NTS has markedly advanced over recent years through the investigation of host interaction with genetically modified mutant *Salmonellae* and through the development of suitable experimental models^15^. After oral entry, *Salmonella* actively invades intestinal epithelial cells, and the SPI-1-encoded type III secretion system (TTSS) provides the effector molecules required for tissue invasion^16^. *Salmonella* can survive in phagocytic and non-phagocytic cells. Intracellular survival requires a second TTSS that is encoded by SPI-2^17^. Much of our knowledge on *Salmonella* pathogenesis came from mammalian host responses to *Salmonella*. Mice have been extensively used as a model for experimental infections. Most work in chicken models has focused on the mechanisms of enteric infection by *Salmonella* Typhimurium (ST) and enteric or egg infection by *Salmonella* Enteritidis^18^. Avian salmonellosis can be classified into three distinct phases, in a pattern similar to that of mammals. Invasion of the gastrointestinal tract marks the first phase of the disease. The second phase is the establishment of systemic infection mainly by intracellular infection of macrophages. In the third phase, infection is cleared by an immune response, the bird succumbs to the infection, or a subclinical carrier state develops^18^. However, differences between mammalian and avian host responses to *Salmonella* prevent accurate comparisons^15^. Little information is available on the pathogenesis of non-host specific NTS, such as SM. In the absence of a suitable model, investigation of SM infection in chickens is pragmatic.

*In vivo* and *in vitro* insights into the pathobiology of SM infection in chicken may help to devise effective control and treatment strategies. To this aim, we investigated for the first time, the pathobiology of SM infection in chickens, dissemination in chicken organs and eggs, and the pathological implications of experimental infection with wild-type SM in specific pathogen free (SPF) chickens. We also investigated cellular SM uptake by chicken peritoneal macrophages, *in vivo* invasion levels by using rabbit and chicken intestinal loops, serum complement killing sensitivity, and the histopathology and immunohistochemistry of infected organs.

## Results

### *Salmonella* Montevideo is more sensitive to chicken complement than rabbit complement

**Figure 1:**
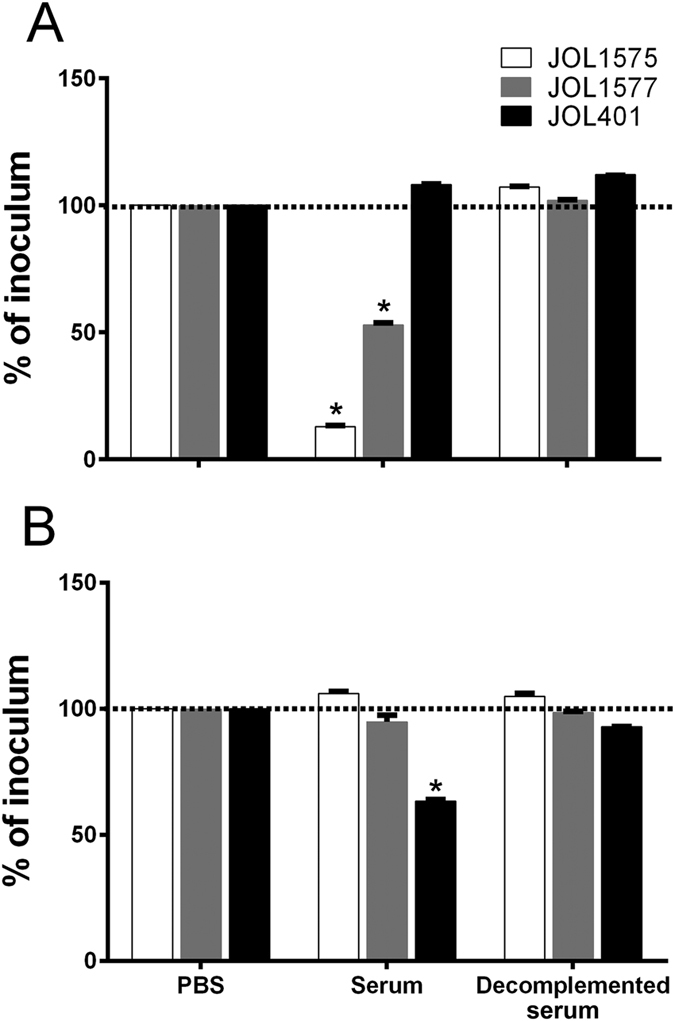
Serum complement sensitivity assay. *S*. Montevideo serotype susceptibility to complement killing was assessed with chicken and rabbit complements. One hour after incubation with complement, viable bacteria were determined by plating on BGA plates. CFU of bacterial strains are represented as percent reduction. (**A**) SM strains were more susceptible to chicken serum than was the reference ST strain. (**B**) SM was relatively resistant to rabbit complement. *P ≤ *0.05;* error bars indicate the SEM.

To evaluate the sensitivity of SM strains to serum complement killing, JOL1575, JOL1577, and reference ST strains were exposed to chicken and rabbit serum complements. The principle behind the test was to determine the sensitivity of the bacteria to complement-mediated bacterial lysis. Fresh serum contains an active complement system that lysed susceptible organisms, but heat treatment for 30 min at 56 °C inactivated the serum complements leading to decomplementation. The effect of bacterial cell lysis by serum complement killing was measured in CFU. SM was more susceptible to complement lysis by chicken serum than was ST (Fig. 1). However, SM was relatively resistant to complement killing by rabbit serum.

### *Salmonella* Montevideo invades chicken intestinal epithelium in a similar pattern to *Salmonella* Typhimurium

**Figure 2:**
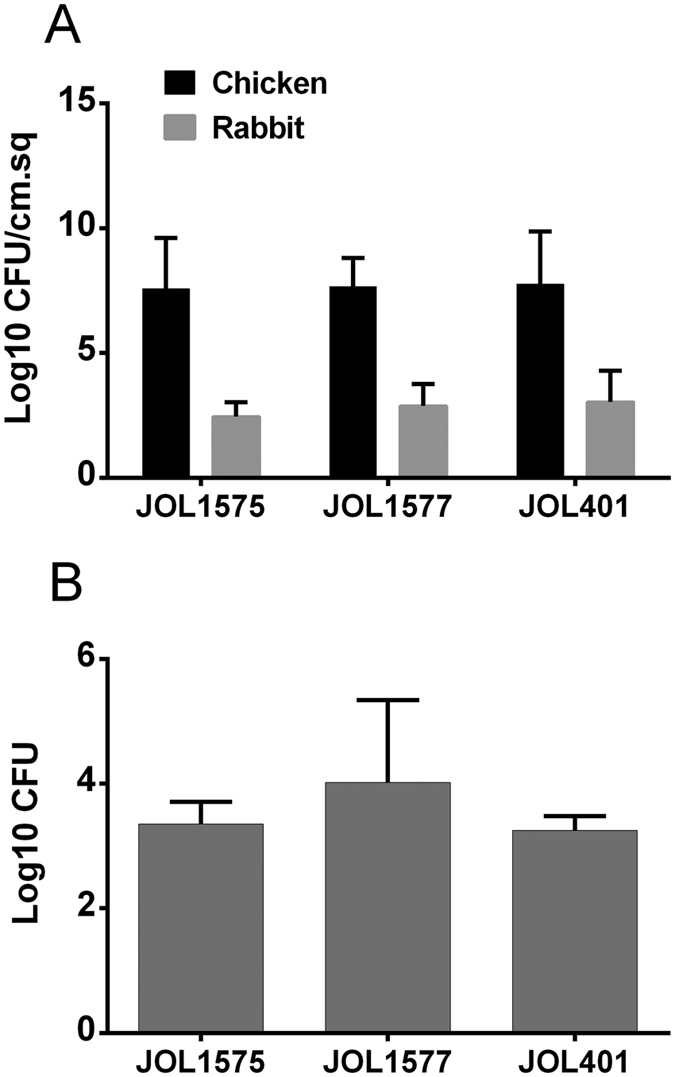
*In vivo* and *in vitro* invasion assay. The ability of SM strains to invade host tissues and cells was investigated with intestinal loop assay and macrophage uptake assay. (**A**) Ligated chicken and rabbit intestinal loops were inoculated with 1 × 10^8 ^CFU of JOL1575, JOL1577, and JOL401 strains. Intracellular bacteria were harvested by cell lysis and plated on BGA plates. Bacterial CFU were obtained for each loop, and the mean log10 CFU/cm.sq ± SEM was determined. The invasion capability did not differ significantly among strains in the chicken or rabbit intestinal models. However, each strain had diminished invasion capability in the rabbit model as compared to the chicken. (**B**) *In vitro* cellular uptake was investigated by using chicken intraperitoneal macrophages. A total 1 × 10^5^ cells were infected with JOL1575, JOL1577, or JOL401 at an MOI of 10:1. Extracellular bacteria were killed with gentamicin antibiotics incubation. Internalized bacteria were harvested by cell lysis 2 hr after inoculation, and total CFU per well were determined. SM strains and ST did not differ significantly on the macrophage uptake assay. Error bars indicate the SEM.

An intestinal loop assay was performed to ascertain the degree of invasiveness of the strains to the intestinal epithelium *in vivo*. The invasiveness of wild-type SM JOL1575, JOL1577, and JOL401 was compared to evaluate the ability of the model to demonstrate differences between strains. The degree of intestinal epithelial invasion by JOL1575 and JOL1577 was highly similar to the reference strain, JOL401. The competence of SM strains in invading the epithelium of chicken intestines was equivalent to that of ST (Fig. 2A). The rabbit intestinal loop model did not show significant differences between the SM and ST strains in terms of intestinal- bacterial uptake. The average log10 CFU of bacteria recovered from the chicken intestinal loop was 7.57, 7.66, and 7.77 from loops dosed with SM JOL1575, SM JOL1577, and ST, respectively. The mean uptake of the rabbit intestinal loop was 2.46, 2.88, and 3.04 from loops dosed with SM JOL1575, SM JOL1577, and ST, respectively (Fig. 2A).

To examine the *in vitro* cellular invasiveness of the strains, macrophage uptake was assessed in chicken primary intraperitoneal macrophages. Both serotypes were efficiently internalized by chicken peritoneal macrophages. No significant difference was observed between JOL1575 and JOL401 in their propensity to taken up by macrophages (Fig. 2B).

### *In vivo* tracking of JOL1575GFP dissemination in chickens

**Figure 3:**
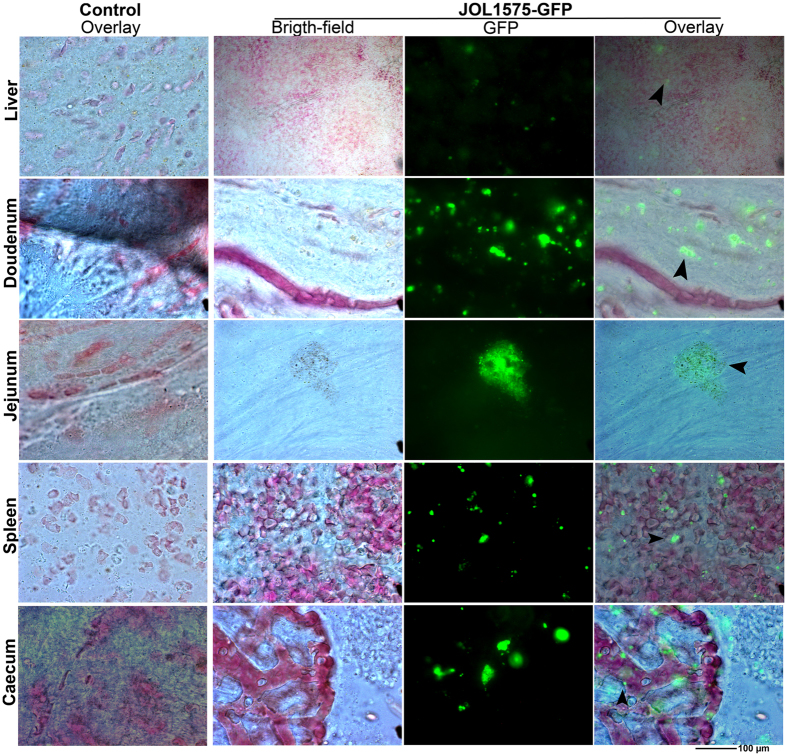
*In vivo* tracking of SM-GFP infection in chicken organs. SM dissemination was tracked with a GFP-tagged SM strain at 48 hr after mock infection. Immunologically mature SPF chickens were inoculated with JOL1575GFP expressing GFPuV which is having a peak excitation at 395 nm and a peak emission at 509 nm. Uninoculated chickens were used as a control to differentiate autofluorescent tissues. Thin tissue biopsy sections were collected from representative organs and observed under a fluorescent microscope. Infection foci were observed in the liver, small intestines, spleen, and cecum of infected birds. The organs of control uninoculated birds had no SM-GFP fluorescence signal. Black arrow heads indicate fluorescent signals emitted by SM-GFP in infected tissues.

Visceral chicken organs affected by inoculation with wild type SM were monitored and tracked with strain JOL1575GFP expressing GFPuV (Supplementary Fig. 1). At 48 hr after inoculation, the spleen and cecum were the main sites of SM bacterial localization. Fluorescent signals from the SM-GFP strains were also detected in the liver, although at a lower intensity than in the spleen or intestinal epithelial tissues. The duodenum and jejunum also revealed infection (Fig. 3). GFP signals were not observed in the crop, lungs, or proventriculus. Reproductive tissues did not show any signs of SM localization or infection. Oviduct and ovarian tissues did not show a JOL1575GFP signal upon fluorescence microscopic examination. *Ex vivo* whole-organ fluorescence images were also acquired, with representative spleens showing fluorescence (Supplementary Fig. 2). No SM-GFP-associated fluorescence signals were observed in organs of control uninoculated birds.

### *Salmonella* Montevideo infection showed minimal pathological lesions in chickens

**Figure 4:**
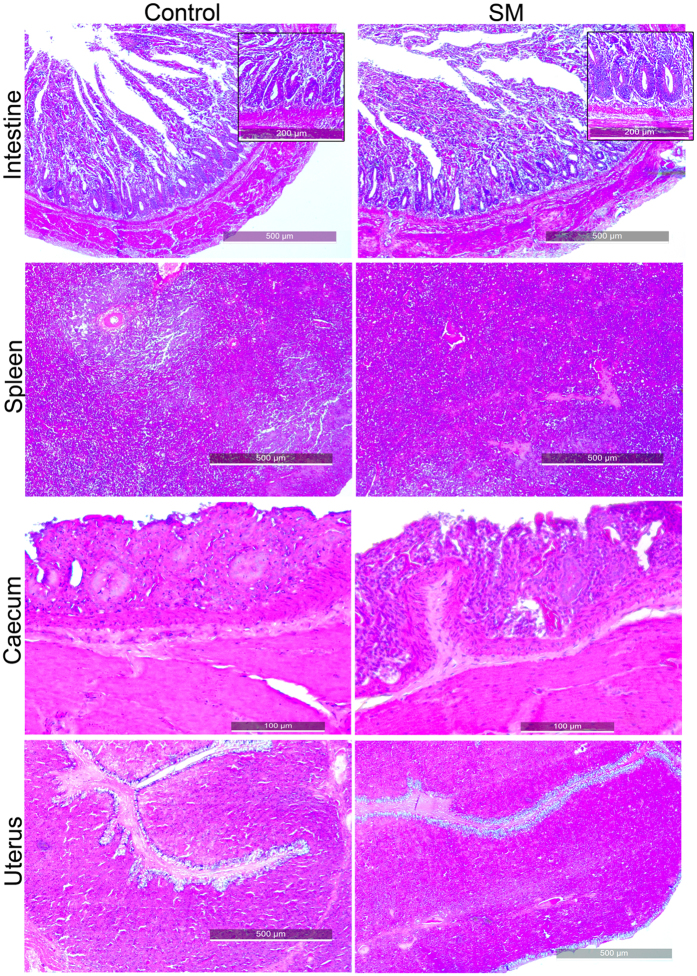
Histopathology of SM-infected small intestine, spleen, cecum, and uterus. Brown nick layers were mock infected with wild-type SM strains. Twenty days after inoculation and establishment of SM infection, birds were euthanized, tissue samples were collected from the small intestine, spleen, cecum, uterus and subjected to H&E-based histopathology. Representative H&E images of organs from mock-infected chickens are shown. On the SM-image panel, no prominent evidence of pathological lesions, such as necrosis, disruption of villi were observed.

**Figure 5:**
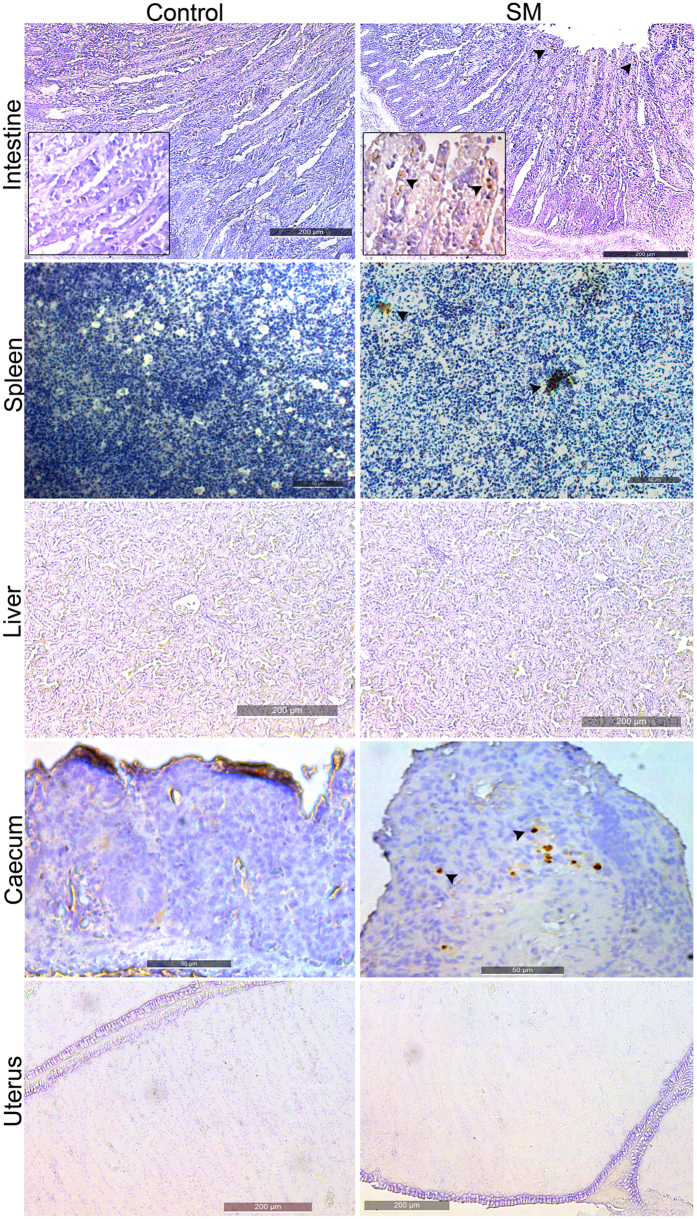
Immunohistochemical analysis of SM infection in chicken. Brown nick layers were mock infected with wild type JOL1575 and JOL1577 strains. Twenty days after inoculation, tissue sections were collected from the liver, intestines, cecum, uterus, and spleen and subjected to IHC analysis. Representative IHC images of chicken tissue sections 20 days after infection with a chicken anti-*Salmonella* polyclonal antibody. *Salmonella* was detected in the apical portion of the intestinal villi, in the splenic parenchyma and caecal mucosa. Sections were visualized with DAB chromogenic substrate and hematoxylin counterstain. Black arrowheads indicate reactive signals specific for *Salmonellae* on the tissue sections.

To gain insight into SM interaction with chicken organs, tissue samples from mock-infected chickens were investigated by using histopathological sections and immunohistochemical analysis. Tissue samples were collected at 20 days after inoculation to allow SM infection to establish in permissive tissues and allow the initial host inflammatory response to subside. No conspicuous tissue damage was observed in histopathological samples of the intestines 20 days after infection (Fig. 4). Although SM bacteria were detected with specific anti-SM antibodies in the villi of the intestine, spleen, and cecum, no disruptive or characteristic damage on the epithelia was observed (Fig. 5). The splenic parenchyma also did not have any conspicuous abnormality, such as lymphocyte depletion or giant cell infiltration. We also investigated the possible involvement of the reproductive tract in internal egg contamination. Immunostaining and histopathology of uterus sections from SM-infected layer chickens did not reveal the presence of SM (Fig. 5). Strains JOL1575 and JOL1577 had no variations in pathological lesions.

### *Salmonella* Montevideo causes internal contamination of hen eggs

The degree of internal egg contamination by SM was studied in immunologically mature brown nick layers, which were monitored for four weeks after inoculation. Immediately after inoculation with wild-type SM, egg production declined drastically (Table 1). The mean egg production was 50.75 ± 0.74, 48.5 ± 1.94, and 46.25 ± 2.09 for control, JOL1575-inoculated, and JOL1577-inoculated groups, respectively. The egg production rate for 4 weeks after inoculation was 72.50%, 69.29%, and 66.07% for the control, JOL1575-inoculated, and JOL1577-inoculated groups, respectively. Of the internal egg contents 1.66% and 2.25% tested positive in the JOL1575 and JOL1577 groups, respectively, in the first week after inoculation (Table 1). No further egg internal contamination was observed during mock infection of layers with SM. However, since SM continued to reside in the host much longer than the study period, sporadic internal egg contamination beyond the experimental time period was not ruled out.

## Discussion

The involvement of live poultry as well as poultry meat and products in transmitting SM infections to humans is well documented^4^,^9^,^19^. Insight into chicken host-pathogen interactions is important not only to elucidate SM bacterial pathogenesis, but also from an epidemiological perspective. To date no significant studies related to chicken SM infection have been reported. In this study, dissemination of SM infection was monitored by using GFP expressing JOL1575-GFP. The main drawback of whole body imaging and tracking is background fluorescence emitted by auto-fluorescing tissues^20^. Background fluorescence may preclude in-depth analysis of GFP signal and could mask the investigation. However, in this study we validated the results by using uninfected control chickens. Thin biopsies encompassing several portions of the internal organs were excised, and micro-tissue sections were prepared for fluorescence microscopy. Experimental SM infection was comparable to *Salmonella* infection in avian species. SM had a similar pattern of intestinal invasion at the early phase of infection. However, the infection was not associated with severe enteritis, hemorrhage, or secretory diarrhea.

Host-species differences in complement activity have long been known and attributed to differences in complement proteins^21^,^22^. Complement killing can be impeded by expression of an outer membrane protein encoded by the resistance to complement killing (*rck*) gene, which is present in some NTS strains and interferes with the formation of the membrane attack complex. However, whole-genome sequences of the SM strains revealed the absence of the *rck* gene. Amino acid homology and sequence alignments of Rck proteins from other *Salmonella* strains showed low similarity with any outer membrane proteins of SM strain (Supplementary Fig. 3). O-antigen (O-Ag) also determines the susceptibility of bacterial cells to complement-mediated killing^23^. The SM and ST display smooth phenotype with complete O-chain length. However, our data revealed differences in susceptibility, SM is more susceptible to chicken serum than to rabbit serum at least with the current isolates tested (Fig. 1). This may explain in part why SM infection of chickens is mild and may be more virulent in rabbits and other mammals, such as humans. However, this hypothesis requires further investigation, evaluating extensively on several SM isolates.

Invasion of the gut epithelium is essential to *Salmonella* pathogenesis, and differences in virulence among serotypes may be related to differences in invasiveness. Both *in vivo* and *in vitro* invasion models have been used^24^, but *in vitro* models may not incorporate all possible invasion pathways^25^. The lack of suitable chicken epithelial cell lines further limits the use of *in vitro* models^26^. In this study, a similar pattern of invasiveness was observed between SM strains and the reference strain, ST, both in chicken and rabbit intestinal loops. In chickens, infection of the GI tract by *S.* Typhimurium results in an influx of heterophils and inflammation whereas non-flagellated *S.* Pullorum and *S*. Gallinarum infections cause only limited inflammation. Based on post mortem examination, the swelling or inflammation observed in intestinal loops of the present study was highly similar between SM and ST infected loops (Supplementary Fig. 4). It is tempting to presume that the flagella of SM may evoke an inflammatory response similar to that of flagellated ST. Moreover, the *avr* gene is absent from SM, which has been suggested to inhibit the inflammatory process, particularly cytokine and chemokine induction, by acting on the NF-kB signaling pathway^27^. However, this hypothesis requires further investigation.

The replication and survival of *Salmonella* inside macrophages is key to the progression of systemic infection in both mammals and birds^28^. *Salmonella* mutants that cannot survive within macrophages are less virulent^29^. The present study indicated that SM and ST have similar patterns of macrophage uptake and survival (Fig. 3). However, the cytotoxic and apoptotic effects of SM strains were not assessed. SM replication and survival may be comparable to those of ST *in vivo*.

*Salmonella* starts invading chickens from the gastrointestinal tract. In this study, we observed that the intestinal epithelium, duodenum, ileum, and jejunum were infected by SM-GFP with no site preference. In the initial stage of infection, spleen, liver, and cecum were affected (Fig. 3). Chicken host specific *S*. Gallinarum induces anemia and septicemia, it further causes gastrointestinal tract hemorrhage, massive inflammatory infiltration, and ulceration of the intestinal wall^30^. Typically 6–10 days after experimental infection, the birds succumbed to the infection^30^,^31^. However, in this study SM-infected chickens had no conspicuous pathological lesions on the internal organs. None of the chickens died due to SM experimental infection. In a separate experiment, we observed that SM persisted in the ceca and spleen of chicken for 31 days after experimental infection.

The pathology of the alimentary tract differs among *Salmonellae* infections^32^. Immuno-histochemical analysis of tissue samples from SM mock-infected chickens revealed little evidence of enteritis (Fig. 4). Few samples had mild neutrophil infiltration due to invading SM pathogens. SM bacteria were detected on the apical portion of the intestinal villi. However, no disruption of villi morphology was evident. Splenic and cecal epithelial cells also harbored bacteria without severe cellular infiltration or signs of chronicity. Liver and uterine sections did not harbor SM or show evidence of inflammation or infiltration. Hence, these findings further indicate that chickens maybe asymptomatic carriers of SM infections. This carrier state may result in excretion of large numbers of bacteria in their feces. They may transmit the pathogen by contaminating water or food sources, thus could be responsible for foodborne epidemics. However, in order to evaluate the gravity of the epidemiological risk factor, it would be necessary to investigate the persistency of SM in chickens and viability of the organism in the environment.

*Salmonella* serotypes frequently transmit infection from hens to eggs and progeny^18^. Systemic infection recurs at the onset of egg laying and may spread to the reproductive tract. This phenomenon is related directly to the physiological and hormonal changes associated with sexual maturity in hens^33^. In this study, eggs were only contaminated during the first week after SM infection. Physiological stress and changes in homeostasis among the layers may be responsible for SM spreading to the eggs, especially during the initial stages of infection. Our *in vivo* tracking and bacterial isolation experiments with SM-GFP did not detect significant SM in the chicken ovaries. This result suggests that SM egg contamination may arise from a hematogenous route, unlike *S.* Enteritidis, which colonizes the reproductive tract and internally contaminates eggs before being laid^34^. Egg contamination may occur sporadically beyond the 4-week study period since the chickens continued to harbor SM in their system. Contaminated eggs produced by infected laying hens are thought to be a main source of human *Salmonella* infection^35^. However, external soiling of eggshells can also lead to subsequent entry into the eggs, so the route of internal egg contamination by a *Salmonella* serovar must be validated. We observed low level of internal egg contamination possibly by hematogenous route. Despite the level of contamination, it may still pose a foodborne illness threat.

In conclusion, we studied the effects of SM infection *in vivo* and *in vitro*. We confirmed the organs targeted by SM infection in chickens by GFP tagged SM. SM sensitivity to chicken serum complement may contribute to attenuated SM infection in chickens, apart from inherent host resistance to the organism. SM infection in chickens is sub-clinical as only minimal morbidity was observed in experimentally infected chickens. Immunohistochemical studies revealed SM in epithelial cells of the intestine, spleen, and cecum; however, the anatomical features had minimal disruptions. Eggs were internally contaminated following mock-infection by SM suggesting a hematogenous route, rather than invasion of reproductive cells, was responsible for transient egg contamination. These findings also indicate that SM infection is asymptomatic in chicken hosts. Chickens carrying SM infection pose a risk of poultry-associated SM outbreaks in humans.

## Materials and Methods

### Bacterial strains

All the plasmids and bacterial strains used in this study are listed in Table 2. The wild-type SM strains used in this study were isolated from chickens in South Korea. The strains were characterized by biochemical testing with the API 20E panel^36^ and genetic profiling^36^ with a special focus on the *Salmonella* pathogenicity island (Table 2). For *in vivo* tracking, a green fluorescent protein (GFP)-tagged SM strain JOL1575GFP, was generated by electro-transforming wild-type SM JOL1575 with the pEcoli-6xHN-GFPuv plasmid (Clontech, US). JOL1575GFP was grown in medium supplemented with 40 μg/mL ampicillin. SM and *Salmonella* Typhimurium (ST) strains were routinely grown on LB broth and LB agar plates aerobically at 37 °C. All bacterial strains were stored at −80 °C in growth medium containing 20% glycerol.

### Ethics Statement

All animal experimental procedures were approved (CBNU2015-00085) by the Chonbuk National University Animal Ethics Committee in accordance with the guidelines of the Korean Council on Animal Care. All chickens and rabbits used in the study were housed and maintained humanely; they were provided water and antibiotic-free food *ad libitum*.

### *In vitro* serum complement sensitivity assay

The serum complement sensitivity of SM strains was ascertained with chicken and rabbit sera using a protocol described earlier^37^. Fresh sera, negative of anti-*Salmonella* antibodies were collected from chickens and rabbits. All the bacterial strains were grown to the late log phase, 1 × 10^3^ CFU/100 μl each of JOL1575, JOL1577, JOL401, and DH5α control cultures were incubated separately with 100 μL of PBS, 100 μL of 50% complemented serum and 100 μL of de-complemented serum for 1 hr at 37 °C. After incubation, each test lot was plated on an LB agar plate and CFU were enumerated after overnight incubation at 37 °C. The data were represented as mean percentage reduction. The non-pathogenic lab strain DH5α was used as an internal control to measure complement activity and was most susceptible to complement killing. Three replicates were made for each assay and the experiment was performed twice.

### *In vivo* intestinal loop assay

The intestinal loop assay was performed on rabbit and chicken models (n = 3 each). The assay was conducted as described previously with minor modifications^26^. Experimental animals were fasted and maintained with water only for 24 hr. They were anesthetized by administering 30 mg of tiletamine-zolazepam (Zoletil 50, Virbac, US) and 11.66 mg of xylazine (Ropum, Bayer, Germany). Anesthesia was maintained with 15 mg of tiletamine-zolazepam and 11.66 mg of xylazine. The animals were covered with a sterile surgical blanket, and chicken abdomens were de-feathered. For chickens, a ventral approach was used while a lateral approach was used for rabbits. After the incision area was disinfected, the abdomen was opened, and the jejunum was carefully exposed. Nine to ten ligated loops were constructed with polypropylene blue monofilament surgery suture (Prolene, Ethicon, US). The loops were 4 cm long and separated by spacer loops to allow excision of the loops and minimize the risk of contamination from adjacent loops. Ligated loops were inoculated with 1 × 10^8^ CFU of JOL1575, JOL1577, or JOL401. After inoculation, the loops were reintroduced into the abdomen, and the abdominal wall was sutured. Two hours later, 600 μL of PBS containing gentamicin (300 mg/mL) was added to each loop. After another hour the subject was euthanized with an anesthetic overdose. Three 1 × 1-cm biopsies were taken from each loop. The biopsies were placed in a 15 mL tube and homogenized with 2 mL of ice-cold PBS with 1% triton X-100 by using a tissue homogenizer (T-10 Basic Ultra Turrax, IKA, Germany) for 4 min. After 10 min, 20-fold serial dilutions of the homogenate were made in PBS, and 100 mL of each dilution was spread on BGA agar. The plates were incubated aerobically overnight at 37 °C. *Salmonella* colonies were confirmed using PCR protocol described earlier^36^, and CFU were enumerated. The data were expressed as mean log10 CFU/cm.sq ± SEM.

### Isolation of intraperitoneal macrophage and invasion assay

Sephadex-elicited abdominal exudate cells were harvested from brown nick layer chickens at 7 weeks of age as described previously^38^. Invasion analysis was performed in triplicate with minor modifications to previously reported method^39^. Five milliliters of 1% sephadex G100 solution was injected intraperitoneally in 5- to 6-week-old chickens. Three days after injection, the animals were fasted for 12 hr and euthanized. The feathers were wetted with 70% ethanol, and the skin was peeled off the ventral abdominal area. The exposed muscle layer was again wiped with ethanol. Fifty to 80 mL of chilled cell culture medium RPMI-1640 was injected into the peritoneal cavity through a 24-gauge needle. The abdomen was massaged gently but thoroughly with the liquid medium still inside. Exudates containing intraperitoneal macrophages were harvested via ventral surgical incision. The harvested cells were pelleted and washed with medium by resuspension and re-centrifugation at 2500 RPM (700 RCF) for 8 min. Intraperitoneal macrophages was resuspended in 1 mL of RPMI-1640, and the total viable cell count determined. Macrophage cells (5 × 10^5^) were seeded on 96-well tissue culture plates (SPL, Korea) and kept at 37 °C overnight in a CO_2_ incubator. The sensitivity of each strain against gentamicin was preliminarily tested. Bacterial invasion was estimated by lysing adherent cells with 100 μL of 1% Triton X in PBS and plating the lysate on LB agar at adequate dilutions.

Macrophage uptake of SM and reference strains was assessed as described earlier^40^ with minor modifications. The experiment was performed by infecting chicken primary intraperitoneal macrophages with a multiplicity of infection (MOI) of 10:1. To initiate infection, 5 × 10^6^ CFU of SM or ST was added in triplicates to macrophage cell cultures. The cell culture plates were centrifuged at 200 g for 5 min. The macrophages were incubated at 37 °C in a 5% CO_2_ atmosphere for 20 min. The culture supernatant was removed with a sterile pipette and replaced with 100 μL of RPMI supplemented with 100 μg/mL gentamicin to kill extracellular bacteria. The macrophages were incubated at 37 °C in a 5% CO_2_ atmosphere for 48 hr. The culture media and monolayers were washed twice with an equal volume of RPMI. The cells were lysed with 0.5% Triton-X after 10 min of incubation. The released bacteria were harvested and plated directly on BGA plates and incubated at 37 °C overnight. *Salmonella* colonies were confirmed using PCR protocol described earlier^36^. The experiment was conducted twice and the data were expressed as mean log10 CFU/mL ± SEM.

### *In vivo* tracking of green fluorescent protein-tagged SM JOL1575-GFP dissemination in chickens

Immunologically mature SPF chickens (n = 4) were inoculated with the tagged SM, JOL1575GFP. Uninoculated chickens were kept as controls. At specified time intervals 48 hr after inoculation, chickens were euthanized and organs were isolated aseptically with great care to avoid internal blood-borne *Salmonella* contamination. Ten representative tissue sections were made from each organ. The tissue slices were compressed between a microslide and coverslip. The sections were observed under a fluorescent microscope (Axio Imager 2, Zeiss., Germany) and images were acquired in both the bright field and GFP spectra (AxioVision, Zeiss, Germany).

### Histopathology and immunohistochemical examination of affected organs

To investigate the colonization and progress of SM infection in chickens, 22-week-old brown nick layer chickens (n = 4) were inoculated with JOL1575 or JOL1577. Twenty days after inoculation, representative tissue samples of the liver, spleen, uterus, intestines, and cecum were collected and stored in neutral buffered 10% formalin. Tissue sections were prepared, deparaffinized, stained with hematoxylin and eosin (H&E), and examined by light microscopy (Axio Imager 2, Zeiss, Germany) and digital imaging software (Axio Vision, Zeiss, Germany).

Immunohistochemical staining of the tissue sample was carried out as described previously^41^ with modifications. Paraffin sections were deparaffinized in xylene and graded ethanol and hydrated to distilled water. Three percent hydrogen peroxide was used to block endogenous peroxidase activity. Heat-induced antigen retrieval was carried out at 100 °C for 30 min in antigen retriever citrate buffer (Sigma-Aldrich, US). Non-specific sites on the tissue were blocked with 5% skim milk followed by overnight incubation with a primary chicken anti-*Salmonella* polyclonal antibody at 1:1000 dilution. After blocking with 10% goat serum, a secondary goat anti-chicken IgY HRP antibody at 1:1000 dilution was added. Each step was followed by washing with PBS for 5 min three times. All incubations were done in a humidified atmosphere. Vectastain DAB (Vector Laboratories, US) was used as a colorigenic substrate. The sections were counterstained with hematoxylin and mounted with MM 24 Mounting Media (Leica Biosystems, Germany) for microscopy.

### Internal egg contamination

Twenty- to 22-week-old layer hens were grouped (n = 10) and inoculated orally with 10^8^ CFU SM wild-type strains JOL1575 and JOL1577. Eggs were collected regularly for 4 weeks, and processed every other day. Eggs were cleaned and soaked in 70% ethanol for 3 min. After the egg surface was completely dry, the egg contents were collected in a 500 mL sterile breaker. An equal amount of buffered peptone water was added to dilute the egg contents. After thorough homogenization with sterile spatula, 200 μL of sample was directly plated on BGA plates. Ten milliliters of Rappaport Vassiliadis broth was added to an equal amount of the sample and enriched at 42 °C for 48 hr. The sample was plated on BGA and the bacterial load was measured. SM-specific primers were used to confirm the *Salmonella*-like bacteria. This experiment was repeated in triplicate.

### Statistical analysis

Statistical analyses were used wherever applicable. Analyses were performed with SPSS 16.0 (SPSS Inc., USA). One-way analysis of variance (ANOVA) and Student’s *t*-tests were used to determine statistically significant differences, with *P* values of ≤ 0.05 or ≤ 0.01 considered significant.

## Additional Information

**How to cite this article:** Lalsiamthara, J. and Hwa Lee, J. Pathogenic traits of *Salmonella* Montevideo in experimental infections *in vivo* and *in vitro. Sci. Rep.* **7**, 46232; doi: 10.1038/srep46232 (2017).

**Publisher's note:** Springer Nature remains neutral with regard to jurisdictional claims in published maps and institutional affiliations.

## Supplementary Material

Supplementary Information

## Figures and Tables

**Table 1:** Egg production and egg contamination with *S*. Montevideo.

Group (n = 10)	Mean weekly egg production^a^	Egg production rate (%)^b^	Weekly rate of egg contamination (%)			
			1^c^	2	3	4
Control	50.75 ± 0.74	72.50	0	0	0	0
JOL1575	48.5 ± 1.94	69.29	1.66	0	0	0
JOL1577	46.25 ± 2.09	66.07	2.25	0	0	0

^a^Average of the total egg productions for each group during 4 weeks ± SEM, standard error of mean.

^b^Egg production rate (%) for 4 weeks = (the total number of eggs laid over 28 days)/(the number of hens × 28) × 100.

^c^Internal egg contents from the birds were tested positive of JOL1575 and JOL1577 in the first week post inoculation.

**Table 2:** Bacterial strains and plasmids used in this study.

Strain/plasmid	Description	Reference
JOL1575	*S*. Montevideo wild type, SPI-1 *invAE*^+^ *hilA*^+^ *avr*^–^; SPI-2, amino acid permease; SPI-3, *mgtC*^+^; SPI4, ABC transporter; SPI5, *pipB*^+^	KVCC-BA1400378
JOL1577	*S*. Montevideo wild type, SPI-1 *invAE*^+^, *hilA*^+^, *avr*^−^; SPI-2, amino acid permease; SPI-3, *mgtC*^+^; SPI4, ABC transporter; SPI5, *pipB*^+^	KVCC-BA1400377
JOL1575GFP	*S*. Montevideo wild type JOL1575 harbouring GFP plasmid pEcoli-6xHN-GFPuv	This study
JOL401	*S.* Typhimurium wild type, reference strain	Lab stock
DH5α	*E. coli* control strain, F^–^ *endA1 gyrA96 deoR nupG purB20* φ80d*lacZ*ΔM15 Δ(*lacZYA-argF*)U169, hsdR17(*r*_*K*_^−^*m*_*K*_^+^), λ^−^	Lab stock
pEcoli-6xHN-GFPuv	Plasmid harbouring GFP gene, AmpR, pBR322, *lacI,* 6xHN-GFPuv, T7 promoter	This study
