# Corrigendum: Pathogenic traits of *Salmonella* Montevideo in experimental infections *in vivo* and *in vitro*

**DOI:** 10.1038/srep46786

**Published:** 2017-05-04

**Authors:** Jonathan Lalsiamthara, John Hwa Lee

Scientific Reports 7: Article number: 4623210.1038/srep46232; published online: 04 07 2017; updated: 05 04 2017

The Acknowledgements section in this Article has been omitted. It should read:

“Acknowledgements

This work was supported by the National Research Foundation of Korea (NRF) grant funded by the Korea government (MISP) (No. 2015R1A2A1A14001011)”.

