# Supplementary material for: Pathogenic traits of Salmonella Montevideo in experimental infections in vivo and in vitro
Source: Sci Rep. 2017 Apr 7;7:46232. doi: 10.1038/srep46232 (PMC5384224; doi:10.1038/srep46232)

**Supplementary**

# **Pathogenic traits of *Salmonella* Montevideo in experimental infections *in vivo* and *in vitro***

Jonathan Lalsiamthara, and John Hwa Lee*

College of Veterinary Medicine and Bio-Safety Research Institute, Chonbuk National University, *Iksan Campus*, Iksan 54596, Republic of Korea

*Corresponding author

E-mail address: johnhlee@jbnu.ac.kr

**Figure S1. Florescent micrograph of JOL1575GFP.** JOL1575GFP SM strain was induced with 1mM of IPTG, fluorescence was confirmed prior to experimental infections.

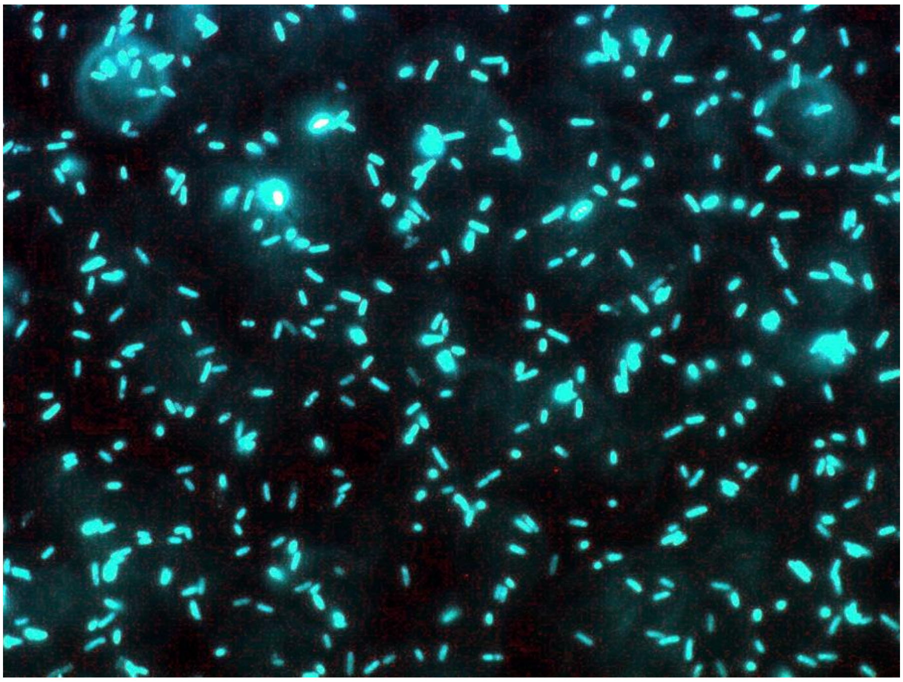

**Figure S2.** Ex vivo Imaging of spleens of experimental birds. GFP signal was observed among birds inoculated with JOL1575GFP SM strain.

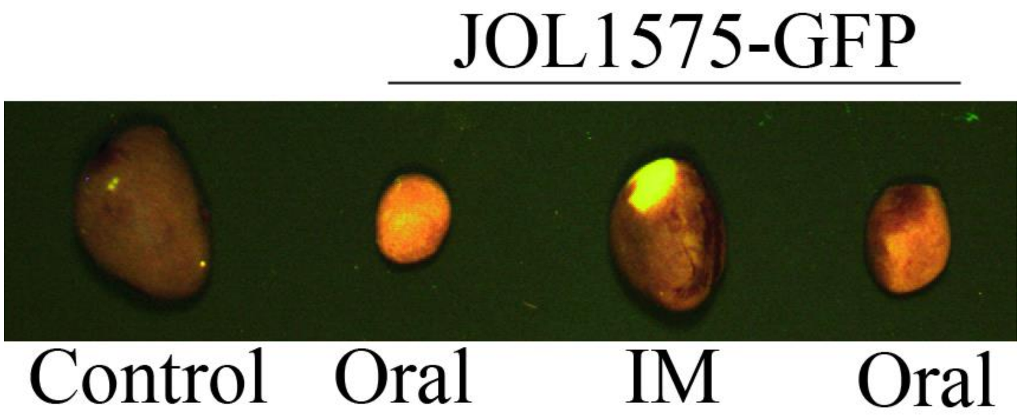

**File S3. Amino acid sequence alignment of Rck protein of *Salmonella* Typhimurium and *Salmonella* Montevideo.** Low degree of homology was observed between Rck protein of ST and SM outer-membrane protein.

hypothetical protein SEEM41H_11051 [Salmonella enterica subsp. enterica serovar Montevideo str. 4441 H]

**Sequence ID:** EHL60847.1 **Length:** 286 **Number of Matches:** 1

See 1 more title(s)

**Range 1: 49 to 280** GenPept Graphics ▼ Next Match ▲ Previous Match

| Score | Expect | Method | Identities | Positives | Gaps |
|---|---|---|---|---|---|
| 127 bits(318) | 1e-34 | Compositional matrix adjust. | 80/237(34%) | 127/237(53%) | 6/237(2%) |

```
Query  88   LTAEKEITGIHGGLSGLTWNPDSRTLFAVTDHPSSVVELDTEGNVLRVIPSDGDHDFEAI  147
            +  +K + G+   LSGLT++ + R LFAV ++P  +V L TEG ++  +P  G HD E+I
Sbjct  49   VIQQKPVAGVKSNLSGLTYSAEDRMLFAVINNPPELVWLTTEGQLVGRMPLQGIHDPESI  108

Query  148  EYLGGNRYALSRERERTLTTHCIDSSTTVLPPATYSLTLDVNRHSDNAGFEGLARGRGEH  207
             + GGN++ +  E++  +    +D     +   +  + L+    + N G EG A
Sbjct  109  AWSGGNQFQIGSEKDGAVYKTQVDIQRGTMQIISM-VKLEGYDKAKNKGLEGTAWDAKNE  167

Query  208  ALMVAQEKKPLRLYVTDRSPDALSMSDSLTHRASLPWFLKDISGLHYDRNNGLLYVLSHE  267
             L  A+E+KP+ +   + S + ++ +      AS    + D+SGL Y      L VLS E
Sbjct  168  RLYAAKERKPIMIKEVEMSKNGITRALPSAITAS----VSDVSGLEYHAPTDSLLVLSDE  223

Query  268  SAVVV-VSGLDGGRKVMSLHRGLCGLRSDIPQAEGITSDDRDTLWIVSEPNLFYRFT  323
            S +++ VS     R  + L     GLR DIPQ EGI  D+ + L+IVSEPNLFY+F+
Sbjct  224  SKMILEVSSEWRVRDRLFLTAEWSGLRDDIPQPEGIAMDNENNLYIVSEPNLFYKFS  280
```

hypothetical protein SEEM315_09874 [Salmonella enterica subsp. enterica serovar Montevideo str. 315996572]

**Sequence ID:** EFY12385.1 **Length:** 286 **Number of Matches:** 1

See 44 more title(s)

**Range 1: 49 to 280** GenPept Graphics ▼ Next Match ▲ Previous Match

| Score | Expect | Method | Identities | Positives | Gaps |
|---|---|---|---|---|---|
| 126 bits(317) | 2e-34 | Compositional matrix adjust. | 80/237(34%) | 127/237(53%) | 6/237(2%) |

```
Query  88   LTAEKEITGIHGGLSGLTWNPDSRTLFAVTDHPSSVVELDTEGNVLRVIPSDGDHDFEAI  147
            +  +K + G+   LSGLT++ + R LFAV ++P  +V L TEG ++  +P  G HD E+I
Sbjct  49   VIQQKPVAGVKSNLSGLTYSAEDRMLFAVINNPPELVWLTTEGQLVGRMPLQGIHDPESI  108

Query  148  EYLGGNRYALSRERERTLTTHCIDSSTTVLPPATYSLTLDVNRHSDNAGFEGLARGRGEH  207
             + GGN++ +  E++  +    +D     +   +  + L+    + N G EG A
Sbjct  109  AWSGGNQFQIGSEKDGAVYKTQVDIQRGTMQIISM-VKLEGYDKAKNKGLEGTAWDAKNE  167

Query  208  ALMVAQEKKPLRLYVTDRSPDALSMSDSLTHRASLPWFLKDISGLHYDRNNGLLYVLSHE  267
             L  A+E+KP+ +   + S + ++ +      AS    + D+SGL Y      L VLS E
Sbjct  168  RLYAAKERKPIVIKEVEMSKNGITRALPSAITAS----VSDVSGLEYHAPTDSLLVLSDE  223

Query  268  SAVVV-VSGLDGGRKVMSLHRGLCGLRSDIPQAEGITSDDRDTLWIVSEPNLFYRFT  323
            S +++ VS     R  + L     GLR DIPQ EGI  D+ + L+IVSEPNLFY+F+
Sbjct  224  SKMILEVSSEWRVRDRLFLTAEWSGLRDDIPQPEGIAMDNENNLYIVSEPNLFYKFS  280
```

**Figure S4. Representative intestinal loop sample.** A. Rabbit intestinal loop, test loops flanked by gap loop. B. Chicken intestinal loop, Arrow head depicting mild swelling in the loop inoculated with SM strain.

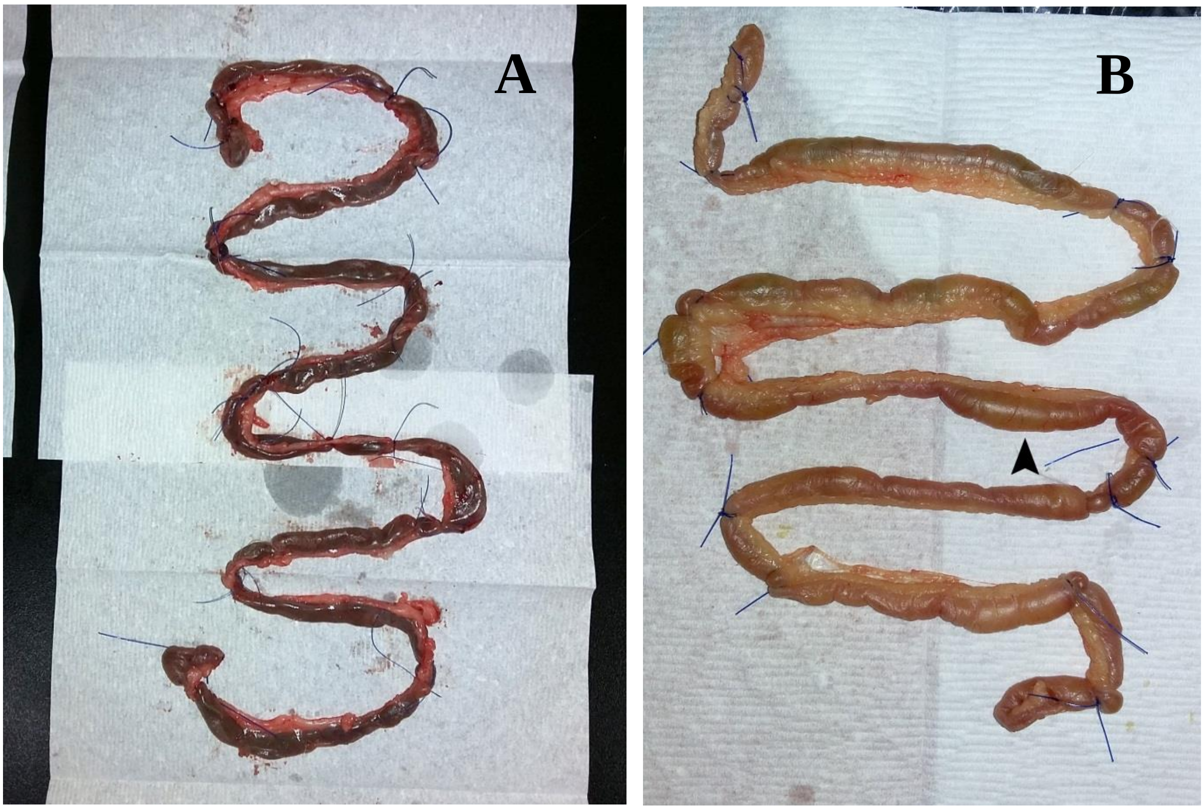

Supplement: Supplementary Information [file srep46232-s1.pdf]
